# Utility of circulating tumor DNA in cancer diagnostics with emphasis on early detection

**DOI:** 10.1186/s12916-018-1157-9

**Published:** 2018-10-02

**Authors:** Clare Fiala, Eleftherios P. Diamandis

**Affiliations:** 1 Department of Pathology and Laboratory Medicine, Mount Sinai Hospital, Toronto, ON Canada; 2 Department of Laboratory Medicine and Pathobiology, University of Toronto, Toronto, ON Canada; 3 Department of Clinical Biochemistry, Mount Sinai Hospital and University Health Network, 60 Murray St. Box 32, Floor 6, Rm L6-201, Toronto, ON MST 3L9 Canada

**Keywords:** Circulating tumor DNA, Biomarker, Cancer diagnosis, Blood test, Early cancer detection, Cancer mutations, Translational omics

## Abstract

Various recent studies have focused on analyzing tumor genetic material released into the blood stream, known as circulating tumor DNA (ctDNA). Herein, we describe current research on the application of ctDNA to cancer management, including prognosis determination, monitoring for treatment efficacy/relapse, treatment selection, and quantification of tumor size and disease burden. Specifically, we examine the utility of ctDNA for early cancer diagnostics focusing on the development of a blood test to detect cancer in asymptomatic individuals by sequencing and analyzing mutations in ctDNA. Next, we discuss the prospect of using ctDNA to test for cancer, and present our calculations based on previously published empirical findings in cancer and prenatal diagnostics. We show that very early stage (asymptomatic) tumors are not likely to release enough ctDNA to be detectable in a typical blood draw of 10 mL. Data are also presented showing that mutations in circulating free DNA can be found in healthy individuals and will likely be very difficult to distinguish from those associated with cancer.

We conclude that the ctDNA test, in addition to its high cost and complexity, will likely suffer from the same issues of low sensitivity and specificity as traditional biomarkers when applied to population screening and early (asymptomatic) cancer diagnosis.

## Background

Circulating tumor DNA (ctDNA) was first described in 1948 [1]. Following the technological advancements that enabled scientists to detect and sequence ctDNA in the blood, various studies and reviews on the utility of ctDNA in cancer have since emerged. The applications of ctDNA can be divided into five broad categories (Table 1), namely prognosis determination, monitoring of treatment and relapse detection, approximation of tumor size and burden, treatment selection, and detection of cancer in asymptomatic individuals. Herein, each category will be briefly discussed to provide the background context to our analysis of the technology required to develop a ctDNA blood test suitable for early cancer diagnosis.

**Table 1:** Applications of ctDNA in cancer diagnostics

ctDNA Application	Summary	References
Prognosis determination	• Absence of ctDNA after surgery is associated with a much better prognosis and smaller chances of relapse • Prognosis determination aids in selecting aggressiveness of treatment as well as determining the necessity for adjuvant therapy; patients at high risk of relapse could receive targeted treatment, while low risk patients are spared unnecessary chemotherapy	[2–7, 10, 21, 29]
Monitoring for treatment efficacy/relapse	• ctDNA can be analyzed through a blood test; this ‘liquid biopsy’ can be repeated more often, enabling consistent monitoring of response to treatment • Raised ctDNA concentrations or increased number of mutations indicate treatment failure/relapse earlier than clinical relapse	[4, 8–22, 29]
Selection of treatment	• Sequencing the ctDNA informs choice of therapy to target specific mutations • Traditional tumor biopsies only allow for the analysis of a certain part of the tumor, which ignores intratumor heterogeneity, while ctDNA analysis provides a more holistic view of the tumor to inform more targeted treatment	[1, 19–21, 28]
Tumor size/disease burden	• Larger amount of ctDNA in blood correlates with advanced tumor stage/greater metastatic burden • Blood testing does not carry the risk of radiation exposure or poor accuracy of imaging; ctDNA can provide a snapshot of disease burden, which can be repeated more often than imaging or traditional biopsies	[19, 21, 23, 24, 30]
Detection in asymptomatic individuals	• Most studies show poor sensitivity, especially for early stage tumors • For small tumors, there is not enough ctDNA present to allow for an accurate test result • However, reliable ctDNA tests for early diagnosis would allow for early intervention and curative surgery and higher cure rates	[16, 20, 25, 30, 32–38]

Using ctDNA to determine prognosis has shown promise across many different cancer types. Striking results were reported in a prospective study of 230 patients with early-stage colorectal cancer, wherein 100% of patients who had detectable ctDNA at the first follow-up visit after tumor resection surgery relapsed within 3 years compared to only 10% of the ctDNA-negative group [2]. Similar observations were reported in longitudinal studies of ctDNA concentrations in lung [3, 4], breast [5, 6], melanoma [7], and ovarian [8] cancers. Knowledge of prognosis can help the clinician make a more informed decision about the aggressiveness and scope of treatment. Additionally, it can aid in ensuring that patients who are more likely to relapse receive adjuvant therapy, while low-risk patients are spared unnecessary treatment [9].

Several investigations have demonstrated the utility of ctDNA monitoring for tumor resistance and treatment success. Traditional tumor biopsies cannot be performed often due to their invasiveness and discomfort, while frequent imaging carries the risk of repeated radiation exposure. However, considerable progress has been made in the technology to sequence and analyze ctDNA. These minimally invasive tests can be repeated frequently, providing constant updates of tumor genetic composition and mutations, and thus informing the best course of treatment [10–13]. Further, they also allow for better monitoring of intra-tumor heterogeneity [9]; unlike traditional biopsies, which only sequence a portion of the tumor, ctDNA provides an overview of all the mutations, allowing for a more targeted treatment. These ‘liquid biopsies’ are now gradually finding their way into the clinic, including FDA-approved EGFR mutation testing for therapy selection [14]. Studies monitoring patients during treatment have shown that lower ctDNA dynamics correlate with better treatment response in colorectal [15], ovarian [16], breast [5], non-small cell lung cancer (NSCLC) [17], and melanoma [18]. Other studies have indicated the potential of ctDNA in detecting resistance, even before its clinical manifestation [5]. For example, in patients with breast cancer, increases in ctDNA concentration provided the earliest indication of impending relapse compared with imaging and other blood-based cancer markers such as circulating tumor cells and cancer antigen 15–3 [5]. An increase in ctDNA was also shown to be more sensitive at screening for relapse than traditional biomarkers in melanoma and NSCLC [19, 20].

A recent investigation highlighting the utility of ctDNA screening for treatment response and resistance was published by Abbosh et al. [21]. In brief, this team sequenced and compared samples from tumor and healthy tissues from the primary surgical resection of patients with early NSCLC to identify the single nucleotide variants associated with cancer. Using this information, they created personalized ctDNA panels for 24 patients, designed to check for relapse by scanning the patients’ blood for mutated ctDNA. These tests were able to detect relapse and resistance in patients 70 days, on average, before tumors became visible on computed tomography scans, with the lead time being over 6 months in four cases. In one patient in this study, sequencing of ctDNA revealed an amplification of the *ERRB2* gene, a cancer promoter that can be targeted by existing chemotherapy medications [21]. Although this application is still being developed, it is proof of principle that ctDNA testing can lead to more personalized treatments. Similar results have also been observed in colorectal cancer, were chemotherapy resistance was shown through detection of resistance-related mutations in circulation months before progression became apparent with imaging [22, 23].

Higher levels of circulating free DNA (cfDNA) have also been associated with greater disease burden and number of metastatic sites [16, 24]. An extensive study of 640 patients with a variety of types and stages of cancer found that the median concentration of ctDNA was 100 times higher in patients with stage IV disease compared to those with stage I disease, providing a basic proportion to estimate tumor size from ctDNA concentration [25]. In their study of early-stage NSCLC, Abbosh et al. [21] were able to develop a more exact metric to estimate tumor size, correlating a higher frequency of mutations in ctDNA or variant allele frequency with a greater tumor volume and finally associating a variant allele frequency measurement of 0.1% with a tumor volume of 10 cm^3^ (27 mm in diameter). Importantly, they also reported that a tumor volume of 10 cm^3^ was required for ideal sensitivity to their ctDNA tests, which is far larger than an early stage/asymptomatic tumor. This presents major sensitivity caveats in using ctDNA for detection in asymptomatic individuals where the tumors would be much smaller. Consequently, the current literature is not supportive of using ctDNA for the detection of small cancers in asymptomatic individuals. In lung cancer, ctDNA is not detectable in all patients with NSCLC [17, 26], showing that the cfDNA quantification/sequencing method is currently limited for lung cancer diagnosis. Abbosh et al. [21] are in agreement with the above, clearly stating that their method is not suitable for asymptomatic early diagnosis. Table 1 summarizes the candidate future applications of ctDNA in the clinic.

Hundreds of millions of dollars have been invested in the lofty goal of developing a blood serum test to detect cancer in asymptomatic individuals. One company, GRAIL, has attracted US$ 900 million in investment capital and accrued funding from Amazon, Johnson & Johnson Innovation, Bill Gates, and Google as well as backing by an impressive number of leading scientists [27]. It is well established in the literature that early cancer detection significantly improves patient outcomes [28]. Thus, if successful, these blood tests will have a tremendous impact on the future of cancer detection and treatment. The tests will involve analysis of the minuscule amounts of cancerous genetic material released into the bloodstream by tumor cells [29]. As ctDNA is generally thought to have the same genetic composition as the tumor it was released from, including all its specific mutations, these tests could provide great insight into the tumor composition [10]. Some companies even hope to create a blood plasma test able to detect the minimal amounts of ctDNA released by asymptomatic or not yet imageable tumors. Detecting cancer at this early stage would mean that tumors would be very small, localized, and far less complex, enabling more effective treatment and a higher cure rate. Thus, in this article, we focus on the detection of very small tumors (less than 10 mm in diameter).

A review of the literature highlights that the utility of using ctDNA for early cancer detection is contested. Therefore, we herein explore, in some depth, the significant difficulties of this approach and the considerable hurdles to the development of a ctDNA blood test for cancer in asymptomatic individuals.

## Relevant calculations based on empirical evidence

ctDNA tests for early diagnosis involve the performance of ultra-deep sequencing of DNA fragments isolated from plasma/serum (liquid biopsy) to identify fragments that have mutations characteristic of malignant cells. These mutant fragments/mutations are considered as unique to malignancy and are not likely to be found in the plasma of normal individuals, which qualifies them as ideal tumor markers [10, 29]. Along with these mutated fragments, the plasma is expected to have otherwise identical, but non-mutated fragments, originating from normal tissues (cfDNA) [2]. Herein, for the sake of discussion, we have assumed that the sample contains a variable mixture of normal and mutant alleles and that the rate of release of these fragments in the circulation is mostly determined by the mass/volume of the tissues, irrespective of their malignant or normal status (Table 2). Similar calculations could be made using other hypothetical scenarios such as the fact that DNA release is 10 times more efficient from tumors compared to normal tissues. Furthermore, we roughly estimated the expected ratio of mutant to normal alleles based on empirical findings from the literature. To screen for a variety of cancers, the tests would need to simultaneously identify a large number (i.e., 50–500 or more) of cancer-associated mutations in plasma. Thus, we assumed that the detection of one mutation would lead to 100% sensitivity and 100% specificity (the best-case scenario). Sensitivity and specificity are addressed further on.

**Table 2:** Assumptions made for cfDNA and ctDNA in patient plasma

Assumption	Justification	References
Fetal DNA in maternal circulation is proportionally related to fetal and maternal weight	It has been documented that, as maternal weight increases, the percent fetal DNA in maternal circulation proportionally decreases	[32]
Circulating tumor DNA and circulating free DNA from normal tissues diffuse into the circulation at roughly equal rates and by similar mechanisms	ctDNA and cfDNA levels are quite variable between normal individuals and patients with cancer; however, as tumor volume increases, the amount of ctDNA also increases, correlating with tumor burden	[4, 16, 24, 25, 32, 48]
In early cancer stages, the amount of ctDNA will not significantly affect the amount of total cfDNA or the circulating genome equivalents	In early stage cancer, the amount of ctDNA is only 0.1% or less, of total cfDNA; thus, it will not significantly increase the number of circulating genomes	[10, 17, 26, 38, 39, 42, 43]
Tumors are spherical and their weight and cellularity are proportional to the volume of the tumor; a tumor of 1 cm^3^ has a wet weight of 1 g and contains approximately 10^9^ cells	Calculations are intended to be approximations in order to estimate the ratio of tumor/normal DNA in the circulation Abbosh et al. [21] reported that a 10 cm^3^ tumor leads to 0.1% ctDNA in the circulation (see text for greater detail)	[21, 39, 43]

In pregnancy, the presence of a foreign body (the fetus within the mother) is a good proxy of tumor presence. Fetal and maternal DNA are distinguishable based on abundance, single nucleotide variants, or epigenetic changes and these differences are now used for prenatal diagnosis of fetal defects such as aneuploidies and genetic diseases [30, 31]. In maternal serum screening programs, performed at approximately 10–20 weeks’ gestation, it has been shown that the amount of total fetal (placental) DNA in circulation is approximately 5–10% of the total DNA (90–95% of which originates from the mother) [32]. The finding that the fetal DNA fraction (the percent of DNA coming from the fetus) is inversely related to maternal weight suggests that similar mechanisms operate during the release of maternal or fetal DNA in the maternal circulation [32], as assumed herein for cancer (Table 2). Considering a fetal/placental unit weight of approximately 0.5 kg at a gestational age of 20 weeks (~ 300 g for the fetus and 170 g for the placenta), the proportional percent DNA for a smaller fetus/placental unit can be roughly estimated by extrapolation (Table 3).

**Table 3:** Ratio of fetal/maternal DNA in maternal circulation

Weight of fetus/placental unit^a^	Percentage fetal DNA in maternal plasma	Ratio of fetal to maternal DNA in maternal circulation	Whole fetal/cancer genome equivalents per 4 mL of plasma^b^	Number of malignant cells in tumor of this size^c^	Likelihood of successful cancer detection
**0.5 kg**	**10**	**1:10**	**1000**	**10** ^**12**^	**High**
100 g	2	1:50	200	10^11^	High
10 g	0.2	1:500	20	10^10^	Moderate
1g^d^	0.02	1:5000	2	10^9^	Low
100 mg^d^	0.002	1:50,000	< 1	10^8^	Very low
10 mg	0.0002	1:500,000	< 1	10^7^	Unlikely
1 mg	0.00002	1:5,000,000	< 1	10^6^	Unlikely

^a^Bold numbers indicate experimental data

^b^1 mL of blood contains approximately 2400 whole genome equivalents in pregnant women and normal individuals [40, 41]

^c^Calculated by extrapolation of data mentioned by Uvili et al. [34]

^d^These ranges have been reported as thresholds for successful detection of cancer based on ctDNA by the most sensitive techniques available to date [29, 60, 61]

The reported amount of circulating DNA in normal individuals and patients with cancer varies widely, likely due to methodological differences and patient characteristics such as cancer stage, vascularization, degree of necrosis, apoptosis, etc. [25, 33]. The range of reported values varies by 1 to 2 orders of magnitude [34–37]; however, most studies cite amounts of cfDNA in normal individuals within the range 1–10 ng/mL (average 5 ng/mL) [10, 38, 39]. Assuming a molecular mass of DNA of approximately 2 × 10^12^, 5 ng of DNA equates to approximately 1500 genomes, which matches well with the amount of DNA previously reported (2400 genomes per mL of plasma in maternal circulation) (Table 3) [40, 41]. According to this data, when the fraction of fetal/cancer DNA drops below 0.01% (one cancer genome admixed with 10,000 normal genomes), then the use of 10 mL of blood (4 mL of plasma) will likely not contain a single fetal/cancer genome for sequencing, thus rendering the diagnosis of cancer impossible due to sampling error.

We also used other reported tumor measures to calculate the approximate amount of cancer or normal DNA in the circulation of patients with small tumors [42]. Table 4 summarizes our calculations, with the bold font indicating experimental data; the rest of the numbers were calculated by extrapolation assuming proportionality between tumor volume and percentage fraction of mutant DNA, as suggested by Abbosh et al. [21]. It is also well accepted that a tumor of approximately 1 cm^3^ in volume has a wet weight of 1 g, contains 10^9^ cells [43], and has an approximate diameter of 1.2 cm (assuming a spherical nodule). It can be seen from this table that, when the fraction of tumor DNA drops below 0.01% (one tumor DNA molecule admixed with 10,000 normal DNA molecules), then 10 mL of blood (4 mL of plasma) will likely contain less than one cancer genome, rendering diagnosis unlikely. Table 4 also shows the likelihood of progression of breast tumors, as reported by Narod [44], and the sensitivity of mammographic screening [45]. If we set an arbitrary clinical requirement of screening to detect cancers that are at least 6% likely to progress and are also now mostly missed by mammography, then a 5 mm diameter tumor would be a realistic and clinically relevant early detection goal. However, this goal is not likely to be met by the suggested ctDNA sequencing technology (Table 4). Other organizations, such as the Ontario Institute for Cancer Research, set goals for the detection of even smaller tumors (as small as 1 mm) [46]. Nevertheless, such over-ambitious goals have to be balanced with the realities of current technologies to avoid over-diagnosis or incorrect results.

**Table 4:** Tumor characteristics reported in the literature or calculated by extrapolation

Tumor diameter, mm	Tumor weight, mg	Tumor volume, mL (cm^3^)	Number of cancer cells	Percentage fraction of mutant ctDNA	Number of cancer genomes per 10 mL of blood	Chance of progression^c^	Mammographic screen sensitivity^d^
27	10,000	10^a^	10,000,000,000	1:1000	6		
12.5	1000	1^b^	1,000,000,000	1:10,000	0.6		
10	500	0.5	500,000,000	1:20,000	0.3	50%	91%
8	250	0.25	250,000,000	1:40,000	0.15	25%	
6	125	0.12	125,000,000	1:80,000	< 0.1		
5	62	0.06	62,000,000	1:160,000	< 0.1	6%	26%
4	31	0.03	32,000,000	1:320,000	< 0.1		
3	16	0.015	16,000,000	1:640,000	< 0.1		
2.4	8	0.007	8,000,000	1:1,300,000	< 0.1		
2	4	0.0035	4,000,000	1:2,600,000	< 0.1		
1.5	2	0.0017	2,000,000	1:5,200,000	< 0.1		
1.1	1	0.0008	1,000,000	1:10,000,000	< 0.1	0.05%	

^a^As reported by Abbosh et al. [21]

^b^As reported by Del Monte [43]

^c^As reported by Cohen et al. [64]

^d^As reported by Erdi [67]

As the empirical data suggests, current methods could predictably detect tumors of between 1 and 3 cm, which are usually at an early stage but frequently present with clinical signs and symptoms. Moreover, tumors of such size are now readily visible through imaging [42, 47].

It is important to underline that both of the modeling scenarios outlined above, using experimental data from pregnancy and NSCLC, predict very similar detectability, pointing to a fractional tumor DNA abundance of 0.01% or higher (Tables 3 and 4).

From the above calculations, it can be concluded that, if a patient has a tumor of 5 mm in diameter, which is considered as an early asymptomatic stage, localized, less likely to progress, and curable, then the ratio of tumor to normal DNA in the circulation will be lower than 1:100,000 (Table 4). If we assume that 1 mL of plasma from a healthy individual contains approximately 3000 whole genome equivalents [39, 40, and our own calculations], then the total amount of whole genome equivalents in the whole blood circulation (approximately 3 L of plasma) will be 9,000,000 (3000 copies multiplied by 3000 mL). Thus, in the whole circulation, only approximately one cancer genome will originate from a 1 mm diameter tumor, with the rest arising from normal tissues (Table 4). Consequently, even if an ultimately sensitive analytical platform able to identify single copies of DNA sequences is used, the likelihood of harvesting one tumor-released DNA fragment from a small (1–4 mm diameter) tumor through a blood draw of 10 mL will be extremely low or non-existent. This would be true even if the total number of genomes released in circulation were to be increased by 10-fold in early cancer. In various cancers, the levels of circulating tumor DNA are higher than in healthy patients, yet, on average, only by 2- to 5-fold [4, 34, 48]; additionally, the differences are expected to be much smaller or non-existent in small and early-stage tumors. The likelihood of this method working consistently is further lowered if we assume the mutant sequence is only present in the sample once. More copies will lead to a more reliable/reproducible estimation.

## Diagnostic specificity and sensitivity issues

**Fig. 1:**
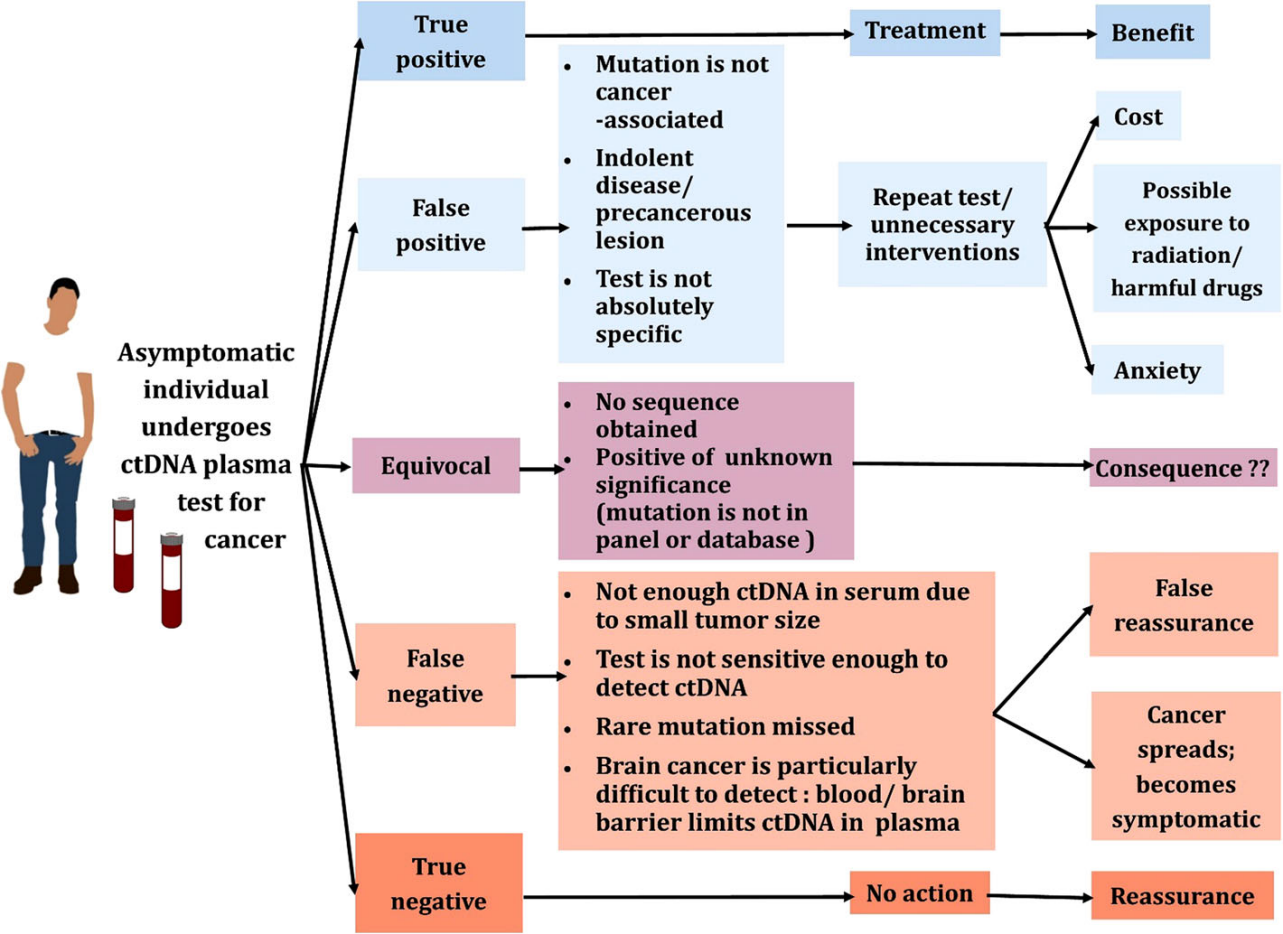
Outcomes and consequences for an asymptomatic individual undergoing a blood serum test for cancer detection

In population screening programs that test asymptomatic individuals, the specificity of the test is of paramount importance, especially if the disease is rare (prevalence < 1:1000) such as in many forms of cancer. For example, if a disease is present in the screened population at a frequency of 1 in 4000 (close to the actual prevalence of ovarian and pancreatic cancer), a population of 100,000 will include 25 affected and 99,975 unaffected individuals. Even if we assume a test’s sensitivity to be close to 100% (so that nearly all affected individuals are captured), a 99% specificity will yield 1000 false positives, with a positive predictive value (PPV) of only 2%. The PPV represents the likelihood of someone who tested positive for a disease actually having the disease. Even at 99.9% specificity, there will still be 100 false positives, yielding a PPV of only 20%. False positive results may lead to undue additional invasive and/or highly expensive tests (Fig. 1). In addition to the specificity prerequisites, several other factors, such as tumor dynamics, influence the outcome of population screening programs, as we and others have previously discussed [49–51]. Screening programs are not very effective for tumors that proliferate quickly (such as invasive breast or pancreatic carcinoma) because patients who originally test negative may go on to test positive with disseminated (thus incurable) disease in the next round. On the other hand, slow-growing tumors, such as prostate cancer, may remain indolent for decades and detecting them in screening programs creates more harm than good through overdiagnosis [52]. It is also imperative for screening programs to prove that those who are screened actually receive tangible benefits such as prolonged disease-specific survival or overall survival.

We have speculated elsewhere that mutated DNA in the circulation may be an ideal tumor marker with superior performance in comparison to traditional biomarkers [53]. However, none of the traditional biomarkers are specific for tumor cells (as opposed to normal cells) since they represent overexpressed or fetal antigens or antigens leaking into the circulation, usually with no relation to tumor biology [54], and it may be incorrect to assume that non-diseased patients have no mutation in their serum/plasma, as recently demonstrated [55–60].

In an important study, Genovese et al. [55] sequenced the DNA of the peripheral blood cells of 12,380 individuals for mutations and followed their health outcomes for 2 to 7 years, arriving to some significant conclusions. Clonal hematopoiesis, namely the formation of a genetically distinct subset of blood cells, was observed in 10% of individuals aged over 65 years but in only 1% of those younger than 50 years. Their investigation revealed that approximately 42% of participants who received a diagnosis of cancer had detectable clonal hematopoiesis with somatic mutations at the time of blood sampling, at least 6 months prior to the first diagnosis. Finally, they found that a portion of some of the genes that are mutated in patients with myeloid cancers are also mutated in healthy individuals and therefore do not cause cancer [55]. Thus, mutations in circulating DNA are not necessary or sufficient for cancer development. This critical finding, namely that not all mutations lead to cancer, is also echoed by Alexandrov et al. [56] in their landmark paper about the mutational rate of clocklike somatic cells.

Furthermore, Schwaderle et al. [57] reported that, among 222 healthy volunteers, one had an alteration in the *p53* gene in cfDNA from plasma (~ 0.5% frequency). Gormally et al. [58] reported mutations with a frequency of 1.2% for *KRAS2* and 3.6% of *p53* genes in plasma DNA from volunteers who were followed for over 6 years and remained cancer free. Fernandez-Cuesta et al. [59] reported an even higher frequency of *p53* mutations in cfDNA from normal controls (~ 11%). Mutations of *p53* in normal individuals were also reported by Newman et al. [60]. It is important to mention here that mutations in *p53* in normal cfDNA may be very difficult to overcome for a diagnostic cancer test since they are the most prevalent genetic alterations in many tumors and drive the sensitivity of such assays, as reported recently by Phallen et al. [61]. These data pose serious challenges for the development of a ctDNA-based screening test. In order to improve sensitivity, ctDNA-based tests must include panels of 100 or more genes, further predictably compromising specificity and reducing confidence of identification due to multiple hypotheses testing [10]. Furthermore, rare variants will likely still be missed in this wide-ranging screening process.

Another new and important discovery is the concept of mosaicism in normal cells and healthy tissue [62]. Neurons have one of the longest lifespans among cells in the body and, as a result, they develop many somatic mutations. These mutations often develop in small populations of adjacent neurons, creating diverse neuronal genomes that are heterogeneous with other regions of the brain. It is increasingly thought that these mutations and cell populations influence neuronal development and function and contribute to various neurodevelopmental disorders. Thus, as the brain develops, subset regions harbor unique single nucleotide variations that are highly specific for a particular region but completely absent in other regions of the brain [62]. This new finding adds yet another challenge in the development of a highly specific cancer test. The Single Cell Sequencing project, which is ongoing, will likely uncover other caveats of individual cell DNA variations that may further complicate the development of a ctDNA blood test for early detection [63].

The most sensitive methods for detecting mutations in ctDNA in the presence of vast amounts of non-mutant DNA are based on the a priori knowledge of mutations that are first found in patient tumors gathered from resection or biopsy. However, in real case scenarios, such mutations will not be known, posing another stress to the assay’s sensitivity. In this respect, Newman et al. [60] developed a highly sensitive assay for detecting mutations in ctDNA without the need for a biopsy. This deep sequencing approach, which incorporated integrated digital error suppression, was able to detect mutant DNA for the EGFR kinase domain admixed with 25,000-fold normal DNA. However, even at these cancer to normal ctDNA ratios, and as predicted in Tables 3 and 4, the likely weight of the detected tumors would be within the 100 mg to 1 g range, well above what is likely needed to detect cancer in asymptomatic individuals.

## Latest results and conclusions

Quality assurance in developing a test for cancer is of paramount importance due to the risk of over- or undertreatment following false positive or negative results, both of which are damaging to patients [51].

This analysis indicates that, apart from technical competence in identifying single nucleotide variations or other changes in circulating DNA, the assumed outstanding specificity of a test derived from these principles is not guaranteed. Even if it were, the value of screening to identify early and curable disease with the suggested method would still have to be assessed. We envision that it will take considerable time before the critical questions raised are answered by prospective studies. The expected outcomes and consequences of ctDNA testing for cancer diagnosis are further summarized in Fig. 1.

**Fig. 2:**
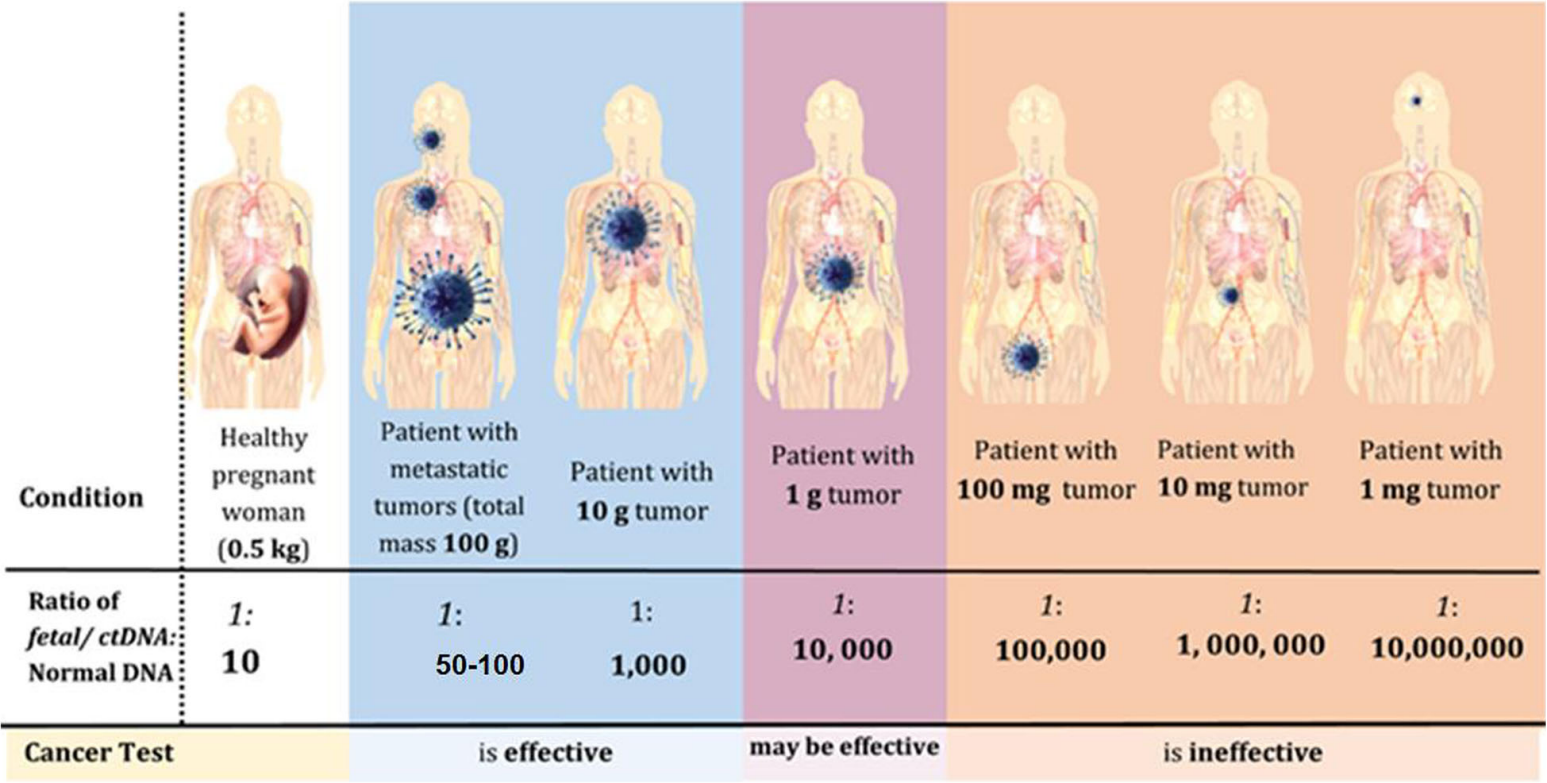
Each patient depicted in this figure has a fetus (far left patient) or a tumor (rest of the patients) of a different mass, decreasing from left to right. Data from Table 4 was plotted and sizes are not to scale. The fetus/tumors secrete DNA into the blood stream in quantities proportional to their masses; the ratio of tumor/fetal DNA (in italics) to total DNA secreted from healthy cells (in bold) is shown underneath a dividing line for each patient. As tumor size decreases, the ratio of circulating tumor DNA to total circulating DNA decreases proportionally. Thus, it becomes increasingly difficult for a test to extract these miniscule amounts of tumor DNA from the rest of the circulating DNA, compromising its effectiveness in detecting small, early stage tumors. For more details see text and Table 4

Theoretical and empirical findings support our conclusion that there is not enough ctDNA in the blood for a sufficiently accurate test result for early or asymptomatic diagnosis (Fig. 2). Additionally, not all mutations signify cancer. However, ongoing research may unveil previously unknown facts that could change our understanding of the advantages and limitations of using ctDNA testing in asymptomatic individuals.

Nevertheless, certain applications of ctDNA look promising (Table 1) such as utilizing it to predict prognosis, monitor treatment efficacy, and development of drug resistance in already diagnosed individuals [10, 21]. Since these tumors are larger and their genetic information is already available from traditional tumor biopsies or resection, ctDNA tests in these situations are poised to provide higher specificity and sensitivity than traditional markers.

Furthermore, while ctDNA testing is being widely researched and developed, it remains very expensive. Abbosh et al. [21] estimated a cost of US$ 1750 to create a personalized assay and perform the tests, yet their panel only targeted 12–30 single nucleotide variants and is significantly below what would be needed to provide a far more comprehensive, diagnostic test. The tests are also time consuming and require specialized skills and equipment; if these were to be performed on a clinical scale, samples would likely have to be shipped to a central location, with a delay in the order of weeks before clinicians could receive the results. In contrast, serum testing for traditional circulating protein markers (such as CEA and CYFRA 21-1 for NSCLC) is far simpler, costs only a few dollars per sample, and can be performed within a few hours. Therefore, in some circumstances classical tumor markers should be preferred, assuming they perform equally well, due to cost, speed, and quality assurance. More research is needed to compare the performance of these traditional biomarkers with that of ctDNA technology to ensure this more expensive technology provides additional information.

## Conclusion

Based on current knowledge and available technologies, ctDNA could be harvested and analyzed to signify cancer only when the tumor weight is in the range of 100 mg to 1 g or has an approximate diameter of ≥ 1 cm. In such cases, the ratio of ctDNA to normal DNA is expected to be within the range of 1:10,000 to 1:100,000. These tumor sizes represent large enough tumors visible by imaging and which are less likely to be curative by radical surgery. Therefore, it would be preferable for the test to be at least 100-fold more sensitive in order to detect tumors of 5 mm in diameter. The major limiting factor in achieving this detection sensitivity is sampling error due to limited blood availability. With such small tumors, the released ctDNA is unlikely to be present even at a single copy in a 10 mL blood draw. Complicating the interpretation is the fact that recent data suggests that mutations in circulating DNA could be found in a significant proportion of normal individuals. In this respect, the new molecular tumor marker, ctDNA, may suffer from the same limitations of classical protein circulating markers, namely low sensitivity and specificity, especially for early detection.

Two very recent studies indirectly confirm our predictions. Phallen et al. [61] claimed early cancer detection with 70% sensitivity and 95% specificity based on ctDNA sequencing, yet all their samples contained more than 0.01% tumor DNA, as we discussed in our recent publication [43]. Additionally, Cohen et al. [64] recently reported a combination of circulating tumor markers and ctDNA for early detection of non-metastatic cancers of the ovary, liver, stomach, pancreas, esophagus, colorectum, lung, and breast cancer. The reported sensitivities ranged from 69 to 98% for ovarian, liver, stomach, pancreas, and esophageal cancer, at > 99% specificity [64]. However, all of their patients were symptomatic at diagnosis.

Recently, the biotechnology company GRAIL performed a highly relevant study, presented at the 2018 ASCO Annual Conference [65, 66], wherein they prospectively collected 1627 samples from 749 controls (no cancer) and 878 patients with newly diagnosed and untreated cancer (20 tumor types of all stages). The overall sensitivity of their blood ctDNA test was between 50 and 90% (stages I–III) but for some cancers (low Gleason grade prostate, thyroid, uterine, melanoma, and renal) the assay had less than 10% sensitivity. Specificity was fixed at 95%. GRAIL claimed that their ctDNA-based blood test detected multiple cancers at various stages with good sensitivity and high specificity, thus being a new, promising multi-cancer screening test. In a separate breast cancer study [66], including 358 patients with invasive breast cancer (mostly stage I–II) and 452 controls, GRAIL also reported, for symptomatically diagnosed breast cancer patients, average sensitivity values of 58%, 40%, and 15%, respectively, in triple negative, HER2-positive/hormone receptor-positive, and HER2-negative breast cancer subtypes, at 95% specificity. However, when patients were classified according to the mode of diagnosis (symptomatic versus screen-detected/asymptomatic), the sensitivities were 44% for symptomatic patients and only 10% for screened-detected/asymptomatic breast cancers.

These newly derived data from GRAIL fully support our notion that this method will be problematic in terms of both sensitivity and specificity for early cancer detection. Diagnostic effectiveness aside, it will also be necessary to address other important issues related to early cancer screening, including over-diagnosis and over-treatment [51]. Currently ongoing studies are expected to shed more light on this highly interesting area of cancer research.

## Abbreviations

cfDNA: circulating free DNA
ctDNA: circulating tumor DNA
NSCLC: non-small cell lung cancer
PPV: positive predictive value
